# Sustainable Production of Insecticidal Compounds from *Persea indica*

**DOI:** 10.3390/plants11030418

**Published:** 2022-02-03

**Authors:** Azucena Gonzalez-Coloma, María Fe Andrés, Rodrigo Contreras, Gustavo E. Zúñiga, Carmen Elisa Díaz

**Affiliations:** 1 Instituto de Ciencias Agrarias, CSIC, Serrano 115, 28006 Madrid, Spain; mafay@ica.csic.es; 2 Laboratorio de Fisiología y Biotecnología Vegetal, Departamento de Biología, Facultad de Química y Biología, Universidad de Santiago de Chile, L.B. O’Higgins 3363, Estación Central, Santiago 917021, Chile; rodrigo.c@thenotcompany.com (R.C.); gustavo.zuniga@usach.cl (G.E.Z.); 3 Centro para el Desarrollo de la Nanociencia y la Nanotecnología (CEDENNA), Universidad de Santiago de Chile, L.B. O’Higgins 3363, Estación Central, Santiago 917021, Chile; 4 Instituto de Productos Naturales y Agrobiología, CSIC, Avda. Astrofísico F. Sánchez 3, 38206 La Laguna, Spain; celisa@ipna.csic.es

**Keywords:** *Persea indica*, aeroponic, ryanodane, antifeedant, insect

## Abstract

In this work, we have investigated the accumulation of ryanoids in different plant parts (leaves, stems and roots) of aeroponically grown *Persea indica* cloned trees (one-year-old cloned individuals) and a selected mature, wild tree. We tested the insect antifeedant (against *Spodoptera littoralis, Myzus persicae* and *Rhopalosiphum padi*) and nematicidal (against *Meloidogyne javanica*) effects of ethanolic extracts from these different plant parts. The HPLC-MS analysis of *P. indica* extracts showed that mature tree (wild) leaves had two times more chemical diversity than stems. Aeroponic plants showed fewer differences in chemical diversity between leaves and stems, with the lowest diversity found in the roots. Ryanodane epiryanodol (**1**) was present in all the plant parts, with the mature stems (wild) containing the highest amount. The aeroponic stems also accumulated ryanoids including **1**, cinnzeylanol (**2**) and cinnzeylanone (**4**). The insect *Spodoptera littoralis* was strongly affected by the stem extracts, while leaf extracts were moderately active. Based on predicted vs. real antifeedant values, we concluded that the ryanoid content (**1** or a combination of **2**, **4** and **1**) explained the antifeedant effects of the stem extracts, while additional components contributed to the activity of the leaf extracts. Therefore, careful individual selection of *P. indica* seedlings should be carried out prior to proceeding with aeroponic cultivation in order to obtain ryanodane-rich stem or leaf extracts with strong antifeedant effects on *S. littoralis.*

## 1. Introduction

The Macaronesian laurel forest, with a unique and endemic species composition, is a plant community comparable to the evergreen, humid forests that were abundant in the Mediterranean during the Paleogene and Neogene. These forests have previously been considered a relict vegetation from the Tertiary. However, they now represent remnants of the Macaronesian Pliocene/Pleistocene forests that modified their distribution areas due to temperature oscillations during the Pleistocene and consist of plant communities originating from European/Mediterranean and tropical regions [1].

One of the dominant species in this forest is *Persea indica* (L.) Spreng., a perennial tree belonging to the Lauraceae family that probably originated from American tropical lineages [1]. *P. indica* has a characteristic defoliated appearance resulting from the seasonal action of the wild rat (*Rattus*). *P. indica* aerial parts contain ryanodane and isoryanodane diterpenes [2,3,4,5], alkene-γ-lactones [6] and avocadofuranes [6,7,8], while the fruits only contain avocadofuranes [6]. 

The ryanodane diterpenes isolated from *P. indica* are toxic to mice [2] and have strong insect antifeedant effects on *Spodoptera litura* and *S. littoralis* [4,9] and moderate effects on *Leptinotarsa decemlineata* [5,10]. The insecticidal activity of these ryanoids is selective [10], acting on a nonneuronal target [11]. A preliminary study on the natural production of ryanoids in wild *P. indica* indicated that the foliar epiryanodol content of mature, naturally growing trees did not show seasonal variation and did not correlate with their nitrogen, water or phenolic content. This diterpene varied among the individual trees and was found to accumulate in the stems, suggesting a genetic-based control [12].

Given the potential of these ryanodanes as bioinsecticides, supercritical and supercritical antisolvent CO_2_ (SC or SAS / CO_2_) selective extraction methods have been developed to separate polar ryanodanes (epiryanodol and related) from alkyl-γ-lactones and related components of low polarity present in *P. indica* aerial parts [13]. However, the fact that this plant is a unique endemic and protected species from the Macaronesian laurel forest represents a bottleneck in the production of ryanodane-based bioinsecticides. Therefore, a method for the sustainable production of *P. indica* biomass is needed. 

Aeroponic cultivation is a soilless production system independent of environmental conditions that involves an efficient use of resources and reduced water consumption. It is carried out in a controlled environment, while spraying the roots intermittently with nutrients of defined chemical composition. It offers complete access to the plants’ aerial parts and roots throughout the production time and provides opportunities to optimize the yield of natural products of interest, thereby facilitating commercial-scale production of bioactive compounds [14]. The aeroponic production of food crops is well known [15], and there are increasing examples of aeroponic production of medicinal plants for the isolation of bioactive secondary metabolites such as whitanolides from *Withania somnifera* and *Physalis* sp. [16], ginsenosides from *Panax ginseng* [17], β-sitosterol from *Cannabis sativa* roots [18], caffeic acid and methyl rosmarinate from *Melissa officinalis* [19] among others, including the sustainable production of medicinal endangered species [20,21]. Aeroponic cultivation of trees has been demonstrated to be a useful tool for eco-physiological studies such as drought pre-conditioning in temperate species [22] or above–below ground carbon fluxes and uptake in tropical species [23,24] and in forestry for vegetative reproduction of rare species [25] and reforestation [26]. However, the aeroponic cultivation of trees for the production of bioactive metabolites is uncommon.

The chemical composition of *P. indica* roots from cloned trees cultivated aeroponically has been recently reported [27]. Alkane-γ-lactones, alkyne-γ-lactones, avocadofurane precursors and *cis*- and *trans*-p-coumarate esters of (−)-borneol have been isolated, along with small amounts of ryanoid diterpenes previously described in the aerial parts [27,28]. Furthermore, among the compounds only found in the roots, (−)-borneol *cis-p*-coumarate and (+)-majorenolide were moderately insecticidal/antifeedant, and (+)-majorynolide was moderately insecticidal and nematicidal [27]. 

In this work, we investigated the accumulation–distribution of insecticidal ryanoids in different plant parts (leaves, stems and roots) of aeroponically grown *P. indica* cloned trees (one-year-old cloned individuals) in contrast with a selected mature, wild tree (>15 m tall). We tested the insect antifeedant (against *Spodoptera littoralis, Myzus persicae* and *Rhopalosiphum padi*) and nematicidal (against *Meloidogyne javanica*) effects of extracts from these different plant parts and correlated their bioactivity with their ryanoid content.

## 2. Results and Discussion

The HPLC-MS analysis of *P. indica* extracts showed that the leaves had two times more chemical diversity than the stems (18 vs. 9 compounds detected) in the mature tree (wild). The immature plants (aeroponic) showed fewer differences in chemical diversity between leaves (12 compounds) and stems (11 compounds), with the lowest diversity found in the roots (9 compounds). Additionally, the most polar fraction of the chromatograms (compounds eluted with water, retention times < 5 min) was higher for the immature (aeroponic) stems (Table 1). 

**Figure 1:**
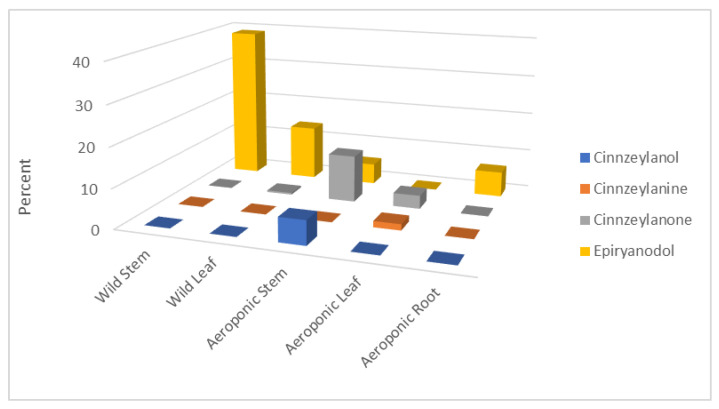
Identified ryanodanes **1**–**4** in *Persea indica* extracts (% extract).

**Figure 2:**
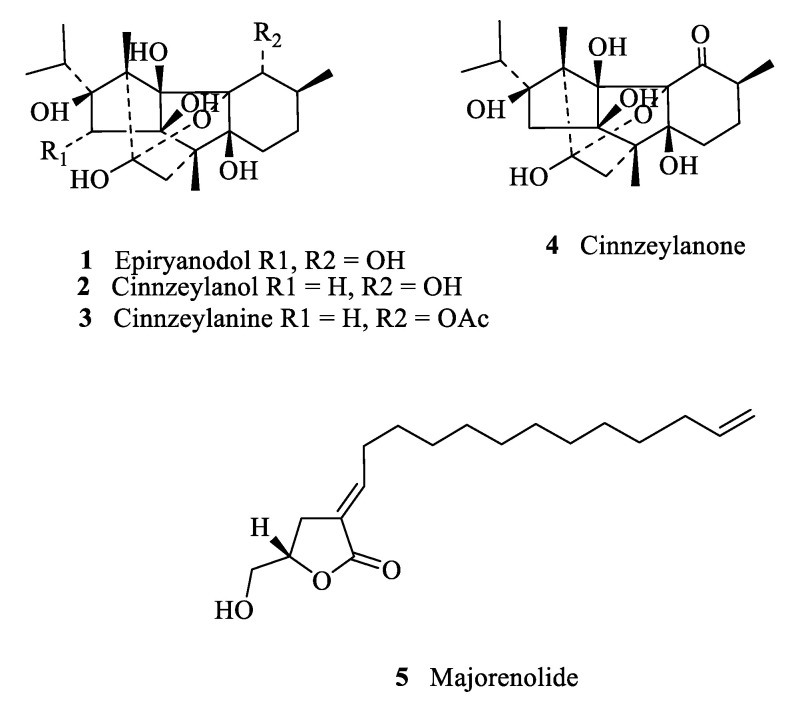
Molecular structures of identified ryanoids **1**–**4** and majorenolide (**5**).

The distribution of the identified ryanoids in *P. indica* extracts (%) is shown in Figure 1. The molecules are shown in Figure 2 and include epiryanodol (**1**) (39% in wild stems, 13.8% in wild leaves, 5.2% in aeroponic stems and 6.4% in aeroponic roots), cinnzeylanone (**4**) (12% in aeroponic stems), cinnzeylanol (**2**) (6.1% in aeroponic stems) and cinnzeylanine (**3**) (1.6% in aeroponic leaves). These results indicate that epiryanodol (**1**) was present in all the plant parts, with the mature stems (wild) having the highest amount. The immature stems (aeroponic) also accumulated ryanoids but with higher molecular diversity.

The major compound detected in the root (28,63%, rt 22.6 min) was not identified. This compound was also present in mature leaf (1.4%), aeroponic stem (4.4%) and aeroponic leaf (2.0%) extracts in smaller amounts (Table 1). Additionally, molecular ions compatible with majorenolide (**5**) (17%, rt 21.23 min, M + 280, m/z M + 2Na + H) were only present in the root extract (Table 1). Majorenolide (**5**), previously isolated from the aeroponic roots of *P.* indica, was moderately antifeedant against the aphids *R. padi* and *M. persicae* (EC_50_ values of 17.6 and 15.8 μg/cm^2^), and cytotoxic to Sf9 insect cells [27].

These extracts were bioassayed for antifeedant effects against three insect species (*S. littoralis, R. padi* and *M. persicae*), and the nematode *M. javanica*. *S. littoralis* was affected by these extracts, with the stems being the most active (mature tree, EC_50_ = 8.5 µg/cm^2^; immature tree, EC_50_ = 12.1 µg/cm^2^). The leaf extracts were moderately active (mature tree, EC_50_ = 36.8 µg/cm^2^; immature tree, EC_50_ = 52.6 µg/cm^2^) while the aeroponic root extract was not active (Table 2).

Based on the relative ryanoid content and the efficient antifeedant doses calculated for each compound identified, we calculated the predicted antifeedant effect (%FI/cm^2^) for each extract (Table 3). The predicted vs. the real antifeedant (% FI) values were similar for both stem extracts (mature and immature) but not for the leaves (2.6 times different). Therefore, the ryanoid content (epiryanodol (**1**) or a combination of cinnzeylanol (**2**), cinnzeylanone (**4**) and epiryanodol (**1**)) explained the antifeedant effects of the stem extracts, while additional components contributed to the activity of the leaf extracts.

Previous phytochemical studies of aerial parts from mature *P. indica* trees showed the presence of ryanodanes other than **1**–**4**: Epicinnzeylanol and epiryanodol monoacetate with antifeedant effects against *S. litura* [9] and *S. littoralis* [10], the isoryanodanes vignaticol and perseanol with antifeedant effects against *S. litura* and *S. littoralis* and inactive indicol [4,10]. Additionally, minor ryanoids such as anhydrocinnzeylanone, garajonone, 2,3-didehydrocinnzeylanone and anhydrocinnzeylanine with antifeedant effects against *S. littoralis* [6] have been isolated from the aerial parts. Therefore, the presence of these minor components in the leaves could explain the difference between the predicted (based on the identified ryanoids) and real antifeedant effects of the extracts.

Compounds previously isolated from the roots include inactive alkene-, alkyne and alkane-γ-lactones, avocadofurane precursors and *cis-* / *trans*-p-coumarates of borneol as the major components, along with minor amounts of ryanodanes such as cinnzeylanone, anhydrocinnzeylanone, cinnzeylanine, cinnzeylanol, perseanol, cincassiol E, perseaindicol, secoperseanol and epiryanodol [27,28]. Among these root components, majorenolide did not have antifeedant effects against *S. littoralis*, had moderately low antifeedant effects against the aphids *R. padi* and *M. persicae* (EC_50_ values of 17.6 and 15.8 μg/cm^2^) and was cytotoxic to Sf9 insect cells [27]. 

A previous study demonstrated that the variations in ryanodol and cinnzeylanol content among *P. indica* mature trees were not seasonal and that they depended on the individual tree sampled [15]. Therefore, careful individual selection of *P. indica* seedlings should be carried out prior to proceeding with its aeroponic cultivation in order to obtain ryanodane-rich stem or leaf extracts. These extracts can be enriched in bioactive compounds by selective SC or SAS / CO_2_ extraction of the biomass (leaves or stems), as previously demonstrated [12].

## 3. Conclusions

The HPLC-MS analysis of *Persea indica* extracts showed that the mature tree (wild) leaves had two times more chemical diversity than the stems. The aeroponic immature plants showed fewer differences in chemical diversity between leaves and stems, with the lowest diversity found in the roots. Epiryanodol (**1**) was present in all the plant parts, with the mature stems (wild) containing the highest amount. The immature stems (aeroponic) also accumulated ryanoids with higher molecular diversity. The insect *Spodoptera littoralis* was strongly affected by the stem extracts, while the leaf extracts were moderately active. Based on predicted vs. real antifeedant values, we conclude that the ryanoid content (epiryanodol (**1**) or a combination of cinnzeylanol (**2**), cinnzeylanone (**4**) and epiryanodol (**1**)) explained the antifeedant effects of the stem extracts, while additional components EC_50_ contributed to the activity of the leaf extracts. Therefore, careful individual selection of *P. indica* seedlings should be carried out prior to proceeding with aeroponic cultivation in order to obtain ryanodane-rich stem or leaf extracts with strong antifeedant effects on *S. littoralis.*

## 4. Materials and Methods

### 4.1. Plant Material

*Persea indica* (L.) Spreng branches were collected from a mature tree (>15 m tall) [15] located in Monte de las Mercedes, Tenerife (28°28′10″ N 16°17′30″ W, 719 m) using a 3 m-long guillotine cutter. Additionally, *P. indica* seedlings donated by the Garajonay National Park nursery, Gomera-Tenerife (28°07′34″ N 17°14′14″ W, 1023 m) were multiplied by stem cutting in perlite-vermiculite with indole-3-acetic acid (IAA). The plants were watered three days/week with a nutrient solution (Nutrichem 20:20:20 N, P, K of Miller Chemical & Fertilizer Corp.; 3 g/L) and maintained in a growth chamber (25 °C, 70% RH with a photoperiod of 16:8 h (L:D) until their transfer to the aeroponic chamber.

### 4.2. Aeroponic Cultivation

The aeroponic cultivation of *P. indica* was carried out as described [27]. Briefly, four cloned plants of 10–15 cm height were selected based on their performance (growth and plant health) for aeroponic cultivation. The plants were transferred to an aeroponic chamber (Apollo 3 system: 33 plants, 240 L, 1750 mm × 1350 mm × 750 mm) located in an environmentally controlled greenhouse (20–30 °C) supplemented with artificial light (16:8, L:D). The roots were sprayed under constant pulverization (every 12 s) with water at 26 °C supplemented with 0.2 g/L Nutrichem (20:20:20 of N:P:K—Miller Chemical and Fertilizer Crop, Hanover, PA, USA) and 0.03 % H_2_O_2_ (33% *w/v* Panreac Química SLU, Castellar del Vallès, Barcelona, Spain) and were cultivated for twelve months in water. Plant material (aerial parts and roots) was collected periodically when tree length was between 20 and 30 cm.

### 4.3. Extraction and Isolation

The mature plant parts were separated, air dried at room temperature in a greenhouse and grounded prior to their extraction in a Sohxlet with ethanol (EtOH) (leaves, 20.7% *w/w* yield; stems, 10.6 % *w/w* yield). The aeroponically grown plants were oven dried (40 °C, 48 h) and extracted with EtOH (72 h) at room temperature (leaves, 21.8% *w/w* yield; stems, 5.2% *w/w* yield; and roots, 16.5% *w/w* yield). The cold extracts were filtered and concentrated in vacuo.

### 4.4. HPLC-MS Analysis

The separation and identification of compounds was performed with LC-MS/MS instruments (Agilent 1200 LC system with G1322A degasser, G1311A binary pump and Agilent 6410 triplequad MS/MS system), employing electrospray ionization (ESI). For separation, a reversed-phased RP-ODS (Agilent Zorbax 150 } 4.6 mm, 5µm) analytical column fitted with an ODS (5µm) precolumn was used. Separation was performed with a gradient elution binary system composed of (A) MiliQ water containing 10 mM ammonium acetate and (B) acetonitrile (ACN) at a flow rate of 0.6 mL/min (0 min, 0% B; 1–5 min, 0%–50% B; 5–10 min, 50%–52% B; 10–20 min, 52%–100%; 20–35 min, 100%–65%), and 20 μL of pre-filtered at 0.45 µm sample was injected. A 10 mM ammonium acetate solution was prepared fresh, filtered through Whatman nylon filters (0.45 µm) using a vacuum system and degassed by ultrasound (60 Hz) for 30 min. Separation was performed at room temperature, mass spectra were scanned over the m/z range of 100 to 1000 in the ESI positive ion mode and analysis of all analytes was carried out in MRM mode. The other operating parameters were as follows: nebulizer gas flow, 8 L/min; drying gas flow, 15 L/min; desolvation line (DL) temperature, 330 °C; and heat block temperature, 400 °C. All chromatographic data were processed using MassHunter software (v 1.10).

Pure compounds previously isolated from *P. indica* were injected in the conditions described above as external standards for identification purposes (see Table 4).

### 4.5. Antifeedant Activity

*S. littoralis, M. persicae* and *R. padi* colonies were maintained at ICA-CSIC, reared on an artificial diet, bell pepper (*Capsicum annuum*) and barley (*Hordeum vulgare*) plants, respectively, and kept at 22 ± 1 °C and >70% RH, with a photoperiod of 16:8 h (L:D) in a custom-made walk-in growth chamber.

The bioassays were conducted as described [29]. The upper surface of *C. annuum* and *H. vulgare* leaf disks or fragments (1.0 cm^2^) were treated with 10 µL of the test substance. The extracts and products were tested at an initial dose of 10 or 5 µg/µL (100 or 50 µg/cm^2^), respectively. A total of 5 to 7 Petri dishes or 20 ventilated square plastic boxes (2 × 2 cm) with 2 sixth-instar *S. littoralis* larvae (> 24 h after molting) or 10 apterous aphid adults (24–48 h old) each were allowed to feed in a growth chamber (until 75% larval consumption of control disks or 24 h for aphids, environmental conditions as above). Each experiment was repeated 2–3 times. Feeding inhibition or aphid settling was calculated by measuring the disk surface consumption (digitalized with https://imagej.nih.gov/ij/ (accessed on 3 January 2022)) [30] or by counting the number of aphids on each leaf fragment. Feeding/settling inhibition (%FI or %SI) was calculated as % FI/SI = [1 − (T/C) × 100], where T and C represent feeding/settling on treated and control leaf disks, respectively. The antifeedant effects (% FI/SI) were analyzed for significance by the nonparametric Wilcoxon paired signed-rank test comparing the consumption/settling between the treatment and control leaf disks. Extracts and com-pounds with an FI/SI > 60% were further tested in a dose-response experiment (1:2 serial dilutions to cover a range of activities between 100 and <50% feeding inhibition with a minimum of 3 doses) to calculate their effective dose EC_50_ (dose to give a 50% settling reduction) from linear regression analysis (% FI/SI on Log-dose, STATGRAPHICS Centurion XVI, version 16.1.02).

Based on the relative ryanoid content (%) and the efficient antifeedant doses calculated for each compound identified, we calculated the predicted antifeedant effect (%FI/cm^2^) for each extract at the standard initial concentration of 100 µg/ cm^2^ as follows:

Predicted %FI (100 µg/cm^2^) = [(%Compound /100) × 50]/EC_50_ (1)

### 4.6. Nematicidal Bioassay

A *Meloidogyne javanica* population maintained on *Lycopersicon esculentum* plants (var. Marmande) in pot cultures at 25 ± 1 °C with 70% relative humidity was used in this work. Egg masses of *M. javanica* were hand-picked from infected tomato roots. Second-stage juveniles (J2) were obtained from hatched eggs by incubating egg masses in a water suspension at 25 °C for 24 h. Bioassays were performed in 96-well plates (BD Falcon, San Jose, CA, USA) as described by Andrés et al. [31]. Extracts and compounds were dissolved in water with a 5% DMSO-Tween solution (0.5% Tween 20 in DMSO), and 5 µL of this solution was added to 95 µL of water containing 90–100 nematodes to obtain an initial concentration of 1 mg/mL per well. Treatments were replicated 4 times. As a control, 4 wells were filled with 95 µL of solvent. The plates were covered to prevent evaporation and were maintained in the dark at 25 °C. After 72 h, the dead J2 were counted under a binocular microscope. The nematicidal activity data were presented as percent dead J2s and corrected according to Schneider-Orelli’s formula [32].

## Figures and Tables

**Table 1:** Chemical profile (HPLC-MS) of *Persea indica* extracts and identified compounds **1**–**5**.

Retention Time (min)	Wild (Abundance %)		Aeroponic (Abundance %)			m/z			Identified Compounds
	Stems	Leaves	Stems	Leaves	Roots				
3.00–5.00	19.61	18.41	39.75	17.83	20.7	Polar compounds eluted with water			
7.30		5.18	6.40	4.33		449	353	266	
7.70		3.36	1.34	7.31		421			
7.73					2.86	153			
7.91					1.48	325	289	417	
7.94	2.53	1.89				397	433		
8.05	3.54	3.63	2.66	2.93		417	381		
8.18	9.59	3.98				399			
8.24			6.12			419	421		Cinnzeylanol **2**
8.35		6.97		14.80	6.96	137			
8.49			2.36			417	483	335	
8.87			4.34			504	505	419	
8.82	1.15	5.57				441	477		
8.94		1.87				431	467	397	
9.00				1.56		433	397		Cinnzeylanine **3**
9.11		1.16		2.65		167			
9.34	6.17	10.70		11.72	7.14	401			
9.53		5.18				195			
9.85		0.44	11.90	3.39		417	381		Cinnzeylanone **4**
10.22		2.11				432	459		
10.31				13.84		486			
10.92				2.21		301	424		
11.03		10.49				179	137		
12.37	5.72	2.69				241	181	113	
21.29					17.16	327	363	443	Majorenolide **5**
22.64		1.38	4.44	2.02	28.63	265			
22.93					3.69	299	147		
23.40	4.02		2.23	8.51	4.95	249	147		
23.69			9.49			461	524	249	
25.43	38.91	13.79	5.25		6.40	339			Epiryanodol **1**
26.31	5.38	3.79				666			

**Table 2:** Insect antifeedant (against *Spodoptera littoralis*) and nematicidal (against *Meloidogyne javanica*) effects of *Persea indica* extracts.

Origin	Part	*S. littoralis*	*R. padi*	*M. persicae*	*M. javanica*
		%FI ^a^	%SI ^a^		% Mortality ^b^
Aeroponic	Leaf	62.30 ± 5.88 * 52.6 (31.2–88.0) ^c^	48.8 ± 10.5	17.1 ± 7.2	4.45 ± 0.60
	Stem	94.85 ±2.85 * 12.1 (7.8–18.7) ^c^	44.9 ± 8.4	21.7 ± 6.5	7.86 ± 3.09
	Root	39.38 ± 7.73 > 100 ^c^	28.5 ± 7.2	22.1 ± 7.2	5.01 ±0.51
Wild	Leaf	89.67 ± 10.13 * 36.8 (28.0–48.0) ^c^	43.7 ± 8.3	42.6 ± 8.7	0.09 ± 2.07
	Stem	98.46 ± 1.49 * 8.5 (2.7–23.9) ^c^	44.8 ± 7.3	41.5 ± 8.8	0.08 ± 0.98

^a^ Percent feeding (FI) or setting (SI) inhibition at a dose of 100 μg/cm^2^. Values are means of 10 or 20 replicates, respectively. Values with asterisk (*) are significantly different according to Wilcoxon paired rank test (*P* < 0.05). ^b^ Percent mortality at a dose of 1.0 mg/mL. Values (%) are means of four replicates (corrected according to Schneider-Orelli’s formula [20]. ^c^ EC_50_ (95% lower-upper confidence limits), concentration needed to produce 50% feeding / setting inhibition

**Table 3:** Ryanoid-based (compounds **1**–**4**) predicted antifeedant effects of *Persea indica* extracts on *Spodoptera littoralis*.

Compound	*S. littoralis*	Predicted %FI ^a^			
	EC_50_ µg/cm^2^ (95% Confidence Limits)	Wild		Aeroponic	
		Stem	Leaf	Stem	Leaf
**1**	0.16 (0.07–0.35)	95.28	32.85	12.5	
**2**	1.26 (0.73–2.20)			2.5	
**3**	0.02 (0.01–0.07)				37.1
**4**	0.04 (0.02–0.09)		5.5	148	29.9
Total predicted %FI		95 *	38 ^ns^	100 *	67 *
Calculated %FI ^b^		98 *	89 *	95 *	62 *

^a^ Predicted %FI (100 µg/cm^2^) = [(%Compound /100) } 50]/EC_50._^b^ From Table 2. * Significant antifeedant effect. ^ns^ No significant effect.

**Table 4:** Molecular weight and LC-MS m/z adducts of compounds isolated from *P. indica*.

Rt (min)	M^+^	m/z			Compound	Reference
8.8	384	419	383		Perseanol	[4]
8.9	384	419	421	383	Cinnzeylanol (**2**)	[2]
9.5	408	461	425		Anhydrocinnzeylanine	[6]
9.6	426	397	433		Cinnzeylanine (**3**)	[9]
10.1	382	417	381		Cinnzeylanone (**4**)	[9]
21.2	280	327	363	443	Majorenolide (**5**)	[27]
22.7	300	121			(−)-Borneol-cis-*p*-coumarate	[27]
25.4	336	487	181		Indicol	[4]
25.4	400	339			Epiryanodol (**1**)	[2,27]

## Data Availability

Data available upon request.
